# Alcohol, White Adipose Tissue, and Brown Adipose Tissue: Mechanistic Links to Lipogenesis and Lipolysis

**DOI:** 10.3390/nu15132953

**Published:** 2023-06-29

**Authors:** Qing Li, Ou Wang, Baoping Ji, Liang Zhao, Lei Zhao

**Affiliations:** 1 Beijing Engineering and Technology Research Center of Food Additives, Beijing Technology and Business University, Beijing 100048, China; 2002020217@st.btbu.edu.cn; 2 National Institute for Nutrition and Health, Chinese Center for Disease Control and Prevention, Beijing 100050, China; wangou@ninh.chinacdc.cn; 3 Beijing Key Laboratory of Functional Food from Plant Resources, College of Food Science & Nutritional Engineering, China Agricultural University, Beijing 100083, China; jbp@cau.edu.cn; 4 School of Food and Health, Beijing Technology and Business University, Beijing 100048, China

**Keywords:** alcohol, adipose tissue, liver, lipogenesis, lipolysis, alcoholic liver disease

## Abstract

According to data from the World Health Organization, there were about 3 million deaths caused by alcohol consumption worldwide in 2016, of which about 50% were related to liver disease. Alcohol consumption interfering with the normal function of adipocytes has an important impact on the pathogenesis of alcoholic liver disease. There has been increasing recognition of the crucial role of adipose tissue in regulating systemic metabolism, far beyond that of an inert energy storage organ in recent years. The endocrine function of adipose tissue is widely recognized, and the significance of the proteins it produces and releases is still being investigated. Alcohol consumption may affect white adipose tissue (WAT) and brown adipose tissue (BAT), which interact with surrounding tissues such as the liver and intestines. This review briefly introduces the basic concept and classification of adipose tissue and summarizes the mechanism of alcohol affecting lipolysis and lipogenesis in WAT and BAT. The adipose tissue–liver axis is crucial in maintaining lipid homeostasis within the body. Therefore, this review also demonstrates the effects of alcohol consumption on the adipose tissue–liver axis to explore the role of alcohol consumption in the crosstalk between adipose tissue and the liver.

## 1. Introduction

Alcohol has long been closely associated with human culture, and in addition to its use as a drug, drinking is also considered a form of relaxation [1]. Although the harmful effects of excessive alcohol consumption in humans are widely understood, alcohol remains a prevalent part of society. The global increase in alcohol consumption has led to a significant rise in morbidity and mortality caused by alcohol-related diseases [2,3]. According to 2018 statistics from the World Health Organization (WHO), alcoholism is responsible for three million deaths worldwide annually, accounting for nearly 14% of the total mortality rate among individuals aged 20 to 40. Additionally, millions of people suffer from disabilities and poor health due to alcohol-related issues [4]. Apart from causing social and psychiatric problems, alcohol consumption is linked to over 200 diseases that affect multiple organs in the body [5]. The organs and systems that can be adversely affected by alcohol consumption include the liver [6], cardiovascular system [7], endocrine system [8], essential nutrient metabolism [9], nervous system [10], and gastrointestinal tract [10]. At the same time, alcohol-related tissue damage is also interrelated, for example, gastrointestinal or liver damage may be related to immune system dysregulation [11]. Chronic alcohol abuse has also been linked to the development of various types of cancer [12].

There are numerous shared pathophysiological mechanisms underlying the tissue and organ damage caused by alcohol consumption, including inflammation, increased oxidative stress, abnormal post-translational protein modifications, impaired anabolic signals, upregulation of catabolic processes, and disturbances in lipid metabolism and signal transduction pathways [12]. Ethanol first affects the mouth after drinking. Ethanol denatures proteins through dehydration, enhances the permeability of the oral mucosa, breaks down the lipid components of the epithelium, and heightens sensitivity to other harmful compounds [13]. The gastrointestinal epithelium is the site of the first-pass metabolism of ethanol. As a result, ethanol can have pathological effects on gut function by compromising barrier integrity, which can trigger local and systemic inflammation as well as microbial dysbiosis [14]. In the stomach, ethanol may cause gastric mucosal damage through different mechanisms [15].

Muscle wasting, which results from an imbalance in protein synthesis and degradation mechanisms, is another potential consequence of alcohol use disorder [16,17]. Alcohol also affects neurotransmission in the brain, resulting in disorders between excitatory and inhibitory synaptic inputs during acute drinking and changes in neural adaptations during chronic drinking [18]. Consuming alcohol increases the risk of developing pancreatitis, but the precise mechanism is unclear. New research suggests that fatty acid ethyl esters (FAEEs), the product of the non-oxidative metabolism of ethanol, may bind to the intracellular membrane, leading to a maladjusted effect [19], and increased lysosome fragility [20]. There is a well-established link between ethanol consumption and several cardiovascular diseases. In men, an increase in alcohol consumption was positively correlated with higher blood pressure levels [21]. In addition, ethanol consumption has been associated with a notable risk of proarrhythmic effects. Nonetheless, low to moderate alcohol intake has been found to protect against critical ventricular arrhythmias and heart arrest [22]. Alcohol dehydrogenase (ADH) in the mammalian lung, along with microsomal cytochrome P450 (CYP2E1) and peroxisomal catalase, can metabolize ingested ethanol [23]. Therefore, alcohol intake is associated with a high incidence of pulmonary inflammatory disease, which increases pro-inflammatory factors, especially interferon gamma (γ-IFN) and interleukin 1-β (IL1-β). There are also growing concerns about the link between alcohol consumption and increased susceptibility to infectious diseases. Alcohol consumption is a recognized risk factor for HIV, tuberculosis, pneumonia incidence, and unfavorable therapy results. Recent studies indicate that heavy and chronic alcohol consumption is linked to a higher risk of contracting COVID-19 and experiencing more severe illness. Chronic or irregular alcohol consumption can increase vulnerability to viral and bacterial pathogens and weaken vaccine efficacy [24].

Among these organs affected by alcohol, the liver is particularly vulnerable since it is the primary site of alcohol metabolism [25]. The metabolic breakdown of alcohol can generate byproducts that can damage the liver, resulting in alcoholic liver disease (ALD). ALD includes a variety of diseases, such as alcoholic fatty liver disease (simple steatosis), alcoholic hepatitis, alcoholic liver cirrhosis, and liver cancer [26]. One of the pathogeneses of ALD is the ectopic deposition of fat in the liver, which is the earliest response to heavy drinking [27]. Therefore, it is necessary to study another tissue affected by alcohol with a series of serious consequences: adipose tissue.

It is increasingly recognized that alcohol has a specific impact on adipose tissue, and alterations in tissue function and metabolism can influence the development of other alcohol-related diseases [28,29].

White adipose tissue (WAT) has the function of storing and distributing energy. Adipose tissue is mainly composed of adipocytes. In adipocytes, adipogenesis and lipolysis are mainly controlled by the hormone pathway [30]: elevated insulin circulating levels suppress fat breakdown and stimulate fat synthesis during feeding, while catecholamine signaling stimulates lipid breakdown during fasting or exercise. Therefore, stored triglycerides (TGs) are in a constant flow state, and energy storage and mobilization depend largely on hormone levels. Adipose tissue serves as a central hub in the regulation of energy balance, supplying energy to various organs and tissues, including the liver, bone, myocardium, pancreas, and brain [31].

There is evidence suggesting that alcohol consumption can lead to impaired adipose tissue function. Long-term intake of alcohol increases fat decomposition, and a small amount of fat production decreases or remains unchanged, which will lead to the loss of adipose tissue and the outflow of fatty acids (FAs) [32], affecting the balance of adipose tissue. Brown fat has been a research hotspot since it was found. Long-term alcohol consumption may increase the thermogenic activity of brown adipose tissue (BAT) [33].

In general, chronic alcohol intake affects both WAT and BAT. The consequent fat malnutrition can result in the accumulation of fat in peripheral organs, exacerbating the pathological effects of chronic alcohol consumption. Therefore, it is of great importance to explore the metabolic disorders and diseases of adipose tissue resulting from alcohol consumption.

This review highlights the latest findings on the mechanisms through which alcohol consumption impacts adipose tissue metabolism and homeostatic function. Following an examination of the impact of alcohol on adipose tissue mass, this review will explore the regulation of lipid balance in lipolysis and lipogenesis.

## 2. Adipose Tissue

In recent years, an increasing number of studies have shown the crucial role of adipose tissue in regulating systemic metabolic control, which extends far beyond its traditional view as a passive energy storage organ [34]. The recognition of the function of adipose tissue as an endocrine organ is now widely acknowledged, and ongoing research is continuously uncovering the significance of the proteins synthesized and secreted by adipose tissue [29]. Furthermore, adipose tissue plays a vital role in glucose regulation by secreting adipokines, such as adiponectin (ADIPO), leptin (LEP), and omentin [35]. Adipose tissue is also important in regulating glucose homeostasis and is the primary site for glucose utilization. The tissue microenvironment of adipose tissue can be shared by many different cell populations, which work together to regulate metabolic activity under physiological or pathological conditions [36].

### 2.1. Basic Concepts of Adipose Tissue

In lean individuals, adipose tissue typically represents about 20–28% of total body mass, with variations largely attributed to biological sex. However, in the obese state, adipose tissue can account for as much as 80% of total body weight [37]. The function of adipose tissue is determined by its distribution and location within the body. Subcutaneous adipose tissue lies beneath the skin and comprises the largest proportion of adipose tissue [38]. Visceral adipose tissue is situated around several organs, especially the kidneys (perirenal adipose tissue), the intestines (mesenteric and omental adipose tissue), the gonads (epididymal and parietal adipose tissue), the vascular system (perivascular or peri-epicardial adipose tissue), and the heart (epicardial and pericardial adipose tissue) [39]. Adipose tissue consists of a diverse array of cells that work together to produce and secrete cytokines, chemokines, and hormones [40]. Adipocytes comprise around one-third of the cells in adipose tissue, while the rest include fibroblasts, endothelial cells, macrophages, stromal cells, immune cells, preadipocytes, and the nervous system cells [41]. 

As the body’s largest energy reserve, adipose tissue is closely tied to energy use and availability. For example, both fasting and inflammation can trigger lipolysis, resulting in the release of FAs from adipose tissue [42]. On the other hand, overconsumption of calories enhances fat accumulation. Thus, adipose tissue mass depends on the long-term balance between adipogenesis and lipolysis. When obesity, diabetes, and metabolic disorders occur, adipose tissue will undergo several disease-related changes, such as increased inflammation, abnormal lipid secretion, hypoxia, increased apoptosis, and oxidative stress [43,44].

### 2.2. Lipogenesis

A report from WHO states that 39% of adults over the age of 18 were overweight and 13% were obese in 2016. Obesity is classified as a chronic disease, and the prevalence of obesity has almost trebled since 1975 [45]. 

Obesity serves as a risk factor for numerous non-communicable diseases, including type 2 diabetes, cardiovascular disease, hypertension, respiratory diseases, certain cancers, and various other diseases and disabilities [46,47]. Obesity results from an imbalance between caloric intake and energy consumption [48]. Adipocytes store surplus energy in the form of lipids via two processes: adipocyte hyperplasia (formation of new adipocytes) and hypertrophy (enlargement of existing adipocytes). Both processes can lead to greater adiposity and promote the release of FAs, peptides, inflammatory cytokines, and adipokines [49]. Therefore, understanding the process of adipogenesis is important for the prevention and treatment of associated disorders.

**Figure 1:**
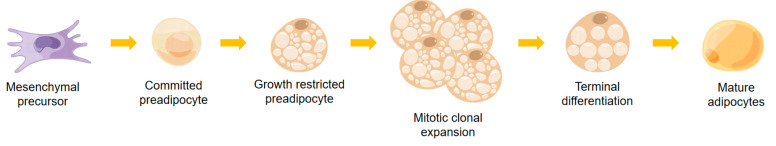
The process of adipogenesis.

As shown in Figure 1, adipogenesis refers to the differentiation of preadipocytes similar to fibroblasts into mature adipocytes with insulin response [50]. This process typically comprises around six stages: mesenchymal precursors, committed preadipocytes, growth-restricted preadipocytes, mitotic clonal expansion, terminal differentiation, and mature adipocytes [51]. For preadipocytes to successfully differentiate into mature adipocytes, they must undergo significant changes in structure and gene expression [50].

Adipogenesis depends on signaling among cells themselves and between cells and their surroundings. Although many details of the molecular mechanisms underlying adipogenesis remain obscure, many factors participating in this process have been recognized. Some stimuli include peroxisome proliferator-activated receptors γ (PPARγ), CCAAT/enhancer binding protein α, β and δ (C/EBPα, C/EBPβ and C/EBPδ), single transcription factors and activators (STATs), transcription factors sterol regulatory element binding protein-1 (SREBP1), insulin-like growth factor I (IGF-l), macrophage colony stimulating factor, FAs, prostaglandins, and glucocorticoids [50,52,53]. Newer studies have pinpointed more factors implicated in this process, comprising krupel-like factors (KLFs), wingless and int-1 proteins (Wnts), and various cyclins [54,55,56]. A series of extracellular signals have also been found to be important, such as bone morphogenetic protein (BMP) and transforming growth factor β (TGFβ), fibroblast growth factor (FGF), notch ligand, proinflammatory cytokines, and hypoxia [57].

### 2.3. Types of Adipose Tissue

Adipose tissue is categorized as white, brown, and beige according to its morphology. In addition, WAT can be further classified according to its location, mainly defined as subcutaneous (located under the skin) and visceral/retinal (located in the abdominal cavity, adjacent to internal organs) tissue. In the majority of healthy individuals, WAT is only present in specific areas. However, in some cases, like obesity and lipodystrophy, WAT may increase ectopically at sites of co-morbid susceptibility such as diabetes and atherosclerosis [57]. BAT is a unique type of adipose tissue with a distinctive morphology and function, as it contains a high density of mitochondria that impart its characteristic brown appearance. In addition, beige adipose tissue represents a novel classification of adipose tissue, distinct from both white and brown adipose tissue.

#### 2.3.1. WAT

Emerging research indicates that in response to particular stimuli, such as exercise, cold exposure, or certain hormones, a type of adipocyte with brown-like characteristics and positive expression of uncoupling protein-1 (UCP1) may appear and be classified as a beige adipocyte [58]. They can accumulate in WAT, often referred to as beige or “brite” adipocytes (brown-in-white).

WAT is a significant source of adipokines, which are peptides that serve as hormones or signaling molecules to regulate metabolism. Up to 40% of genes expressed in adipose tissue are not yet well-characterized, and 20–30% of these unknown genes may encode for secreted proteins [59]. Proteins secreted by adipose tissue have been shown to have multiple functions related to pro-inflammatory cytokines, immunity, fibrinolytic system, angiotensin system, lipid metabolism and transport, and steroid-metabolizing enzymes. Adipose tissue is also considered to be an important endocrine organ that produces adipokines [60]. Proteins secreted by adipose tissue exert effects on the immune, inflammatory, nervous, cardiovascular, reproductive, hematopoietic, and skeletal systems. Adipose tissue is also considered to be an important endocrine organ that produces adipokines [60,61].

#### 2.3.2. BAT

Adipose tissue appears brown stemming from the fact that it is more vascularized and contains more mitochondria which have cytochromes that give the color. The adipocytes that make up BAT are multi-compartmental; these cells are polygonal and range in size from 15 to 50 μm [62]. BAT has the same progenitor cells as skeletal muscle, that is, brown adipocytes originate from precursor cells of small cell tissue rather than white adipocytes [63]. 

BAT differs from WAT in its role as a heat generator, rather than an energy store, through the process of energy expenditure [64]. To better regulate body temperature, brown adipose tissue is distributed in the superficial and deeper layers of the body. Superficially, BAT is distributed in the interscapular, cervical, and axillary regions, while more deeply, it is present in the perirenal, periaortic, inguinal and pericardial BAT [63]. BAT is particularly evident in the neonatal period [65]. Cold exposure and overconsumption of food stimulate the growth and metabolic activity of BAT, which declines with advancing age [66]. In some cases, for example, higher concentrations of thyroid hormones, bile acids, natriuretic peptides, and retinoids have been shown to increase the abundance of brown adipocytes within WAT [67]. 

#### 2.3.3. Beige Adipose Tissue

New research indicates that a beige brown adipocyte-like adipocyte with positive UCP1 expression may be induced by various stimuli, including exercise, exposure to cold or certain hormones [58]. They can accumulate in WAT, often referred to as beige or “brite” adipocytes, which exhibit characteristics of both brown and white adipocytes.

Despite sharing morphological features with brown adipocytes, such as the presence of multiple lipid vacuoles, beige adipocytes are anatomically distinct and located differently [68]. Beige adipocytes are predominantly situated in the subcutaneous regions of WAT, in contrast to brown adipocytes, which are primarily located in the aforementioned superficial regions [69,70]. 

Brown and beige adipocytes originate from separate embryonic precursors [71]. Studies using transgenic mice indicate that beige adipocytes arise from the Myf5-negative lineage [72]. Despite ongoing debate regarding their precise origin, two hypotheses have been put forward: the first proposes that beige adipocytes arise from white adipocyte precursors that undergo transdifferentiation in response to environmental stimuli, such as cold exposure. The second suggests that mature white adipocytes can undergo transdifferentiation through contact with appropriate stimuli [73]. Finally, both mechanisms may be correct, that beige adipocytes are produced through distinct pathways depending on the environment, genetic background, and adipocyte location [74].

## 3. Effects of Alcohol Consumption on WAT

**Figure 2:**
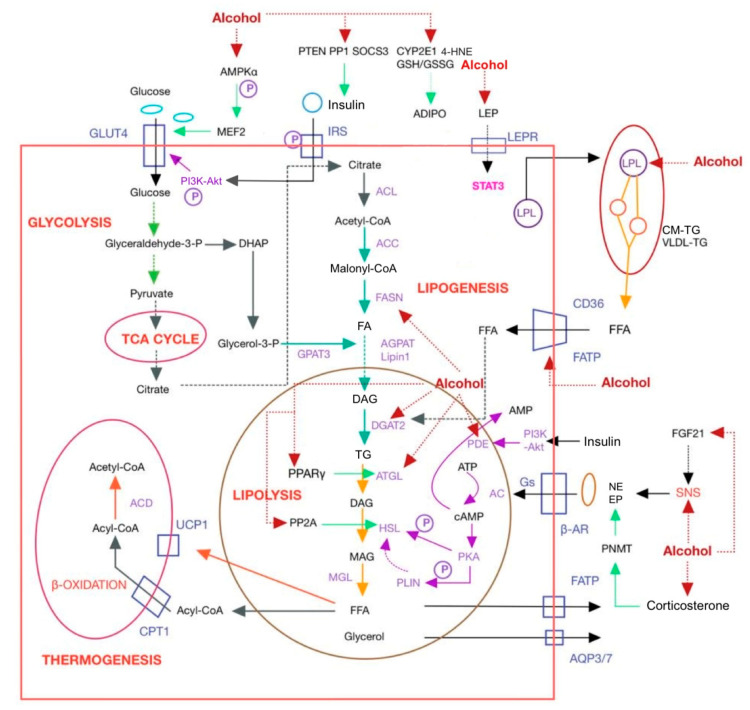
Mechanism of alcohol affecting adipose tissue. AC, adenylate cyclase; ACC, acetyl coenzyme A carboxylase; ACD, acyl-CoA dehydrogenase; acetyl-CoA, acetyl coenzyme A; ACL, ATP citrate lyase; ADIPO, adiponectin; ADIPOR, ADIPO receptor; AGPAT, 1-acylglycerol-3-phosphate acyltransferase; AMPK, AMP-activated protein kinase; AQP, aquaporin; β-AR, β-adrenergic receptor; ATGL, adipocyte triglyceride lipase; CD36, cluster of differentiation 36; CM-TG, chylomicron-triglyceride; CPT1, carnitine palmitoyltransferase 1; CYP2E1, cytochromeP450; DAG, diacylglycerol; DGAT, diacylglycerol acyltransferase; DHAP, dihydroxyacetone phosphate; EP, epinephrine; FA, fatty acid; FASN, lipase fatty acid synthase; FATP, fatty acid transport protein; FGF21, fibroblast growth factor 21; Glyceraldehyde-3-P, glyceraldehyde-3-phosphate; CM-TG, chylomicron-triglyceride; GLUT4, glucose transporter protein 4; GPAT3, glycerol-3-phosphate acyltransferase 3; Gs, GTP-binding protein; GSH/GSSG, glutathione/oxidized glutathione; 4-HNE, 4-hydroxynonenal; HSL, hormone sensitive lipase; IRS, insulin receptor substrate; LEP, leptin; LEPR, leptin receptor; LPL, lipoprotein lipase; MAG, monoacylglycerol; Malonyl-CoA, malonyl coenzyme A; MEF2, myocyte enhancer factor 2; MGL, monoacylglycerol lipase; NE, norepinephrine; PDE, phosphodiesterase; PI3K-Akt, phosphatidylinositol-3-kinase-protein kinase B; PKA, protein kinase A; PLIN, lipid droplet-coated protein; PNMT, phenylethanolamine N-methyltransferase; PP1, protein phosphatase 1; PP2A, protein phosphatase 2A; PPARα, peroxisome proliferator activated receptors α; PTEN, phosphatase and tensin homolog; RXR, retinoid X receptor; SNS, sympathetic nervous system; SOCS3, suppressors of cytokine signaling 3; STAT3, signal transducer and activator 3; TG, triglyceride; UCP1, uncoupling protein-1; VLDL, very low density lipoprotein.

In rats exposed to alcohol, adipose tissue mass decreased [75] and glucose uptake by adipocytes reduced [76], while FAs uptake by hepatocytes increased [77]. This results in an excess of FAs being transported to the liver as triglyceride deposits. Long-term ethanol consumption disturbs the lipid homeostasis of the adipose tissue–liver axis, leading to the accumulation of free fatty acids (FFAs) in both hepatocytes and mesenteric adipose tissue, and thus, triggering metabolic disorders [78]. Hence, it is imperative to comprehend the mechanisms by which ethanol influences lipid synthesis and catabolism in adipose tissue to prevent and treat alcohol-related ailments. Ethanol affects lipid synthesis and lipolysis by influencing various factors in the adipose tissue of rodents and humans, which are shown in Table 1 and Figure 2.

### 3.1. Alcohol Consumption and Lipolysis in Adipose Tissue

#### 3.1.1. Lipolysis

In instances of low metabolic fuel and/or heightened energy demand, such as during periods of fasting, physical activity, and exposure to cold temperatures, adipocytes engage in the catabolic process of lipolysis to mobilize their triglyceride stores and supply peripheral tissues with fuel [91]. The hydrolysis of triglycerides by lipase, known as lipolysis, is a tightly controlled biochemical process that generates glycerol and FFA through enzymatic hydrolysis [34]. Then, aquaporin-3 (AQP3) and aquaporin-7 (AQP7) promote glycerol efflux from adipose tissue and FFA are transported under the action of FA transport proteins (FATPs) [92,93]. Adipocyte triglyceride lipase (ATGL), hormone-sensitive lipase (HSL), and monoacylglycerol lipase (MGL) collaboratively catalyze the stepwise hydrolysis of triglycerides (TG) into diacylglycerol (DAG) and monoacylglycerol (MAG). Each step will release an FFA, and the last step is MGL to release the glycerol backbone of the last FFA. These decomposition products can be re-esterified in adipocytes or released into circulation for use by other tissues [80], such as the liver for gluconeogenesis (glycerol) and oxidative phosphorylation of muscle or other oxidized tissues [94].

Lipolysis of adipose tissue is a finely controlled process [95], which is regulated by various hormones, including adrenocorticotropin hormone (ACTH), epinephrine (EP), norepinephrine (NE), and insulin [96].

#### 3.1.2. β-Adrenergic Receptor Pathway

Catecholamines (EP and NE) are terminal mediators of the sympathetic–adrenergic system, primarily secreted from the adrenal medulla through the sympathetic nervous system (SNS) activation, which are thought to be important regulators of lipolytic activity in major extra-lipidic signal [97]. SNS is activated by sending signals in the brain through FGF21 [98]. Catecholamines are known to exert their physiological effects through binding to β-adrenergic receptors (β-ARs) (subtypes β-1-3-ARs), which are predominantly expressed in adipose tissue [99]. After binding to ARs in adipose tissue, catecholamines stimulate the activity of the stimulatory GTP-binding protein (Gs) [100], increasing intracellular cyclic adenosine monophosphate (cAMP) concentrations. cAMP can be converted by ATP under the action of the adenylate cyclase (AC) [101]. 

Based on these facts, it has been found that chronic alcohol abuse causes lipolysis by increasing SNS activity and stimulating the release of circulating NE and EP, thereby activating the β-AR. Alcohol exposure leads to an approximately four-fold increase in EP release and an approximately two-fold increase in NE release in mice. This is consistent with the clinical observation that shows an increase in blood catecholamine levels with alcohol consumption [86]. In addition, a positive correlation was observed between plasma EP concentrations and mRNA levels of phenylethanolamine N-methyltransferase (PNMT), the main enzyme involved in adrenal EP synthesis. The activity of PNMT is heavily influenced by adrenal glucocorticoids. To investigate further, plasma corticosterone levels were measured in WT mice, and it was found that alcohol exposure significantly increased these levels. This observation provides an explanation for the elevated plasma EP levels [87]. Elevated EP and NE activate lipolysis by promoting β-AR activation, but no differences were found in β-AR expression induced by chronic overeating alcohol exposure [102].

However, a study conducted in vitro showed very different results. Chronic ethanol exposure affects the G-protein signaling pathway associated with β-ARs in cells. During the experiment, for a period of 4 weeks, rats were provided with a liquid diet that consisted of 35% of the total calories in the form of ethanol. Lipolysis was measured in adipocytes that were isolated from epididymal fat by glycerol release (with or without agonist) within 1 h. Ethanol feeding reduced β-AR-stimulated lipolysis [103]. The initial peak of cAMP accumulation was inhibited following ethanol consumption, and the basal cAMP concentrations in the adipocytes, did not differ from the control group [104].

The impairment of β-AR signaling in adipocytes due to chronic ethanol consumption occurs at least at two distinct sites. Firstly, chronic ethanol consumption leads to an increase in the activity of phosphodiesterase 4 (PDE4), resulting in reduced cAMP accumulation [105]; secondly, protein kinase A (PKA)-mediated phosphorylation of perilipin A and HSL, two proteins localized to the lipid droplets of adipocytes, is impaired. As a result, ethanol inhibits the β-adrenergic stimulation of lipolysis [104]. Since the available findings are conflicting on the effects of ethanol feeding on β-AR-stimulated lipolysis [106], there may be other reasons for ethanol-stimulated lipolysis.

#### 3.1.3. Endoplasmic Reticulum Stress Pathway

One of the identified causes is endoplasmic reticulum (ER) stress. Recent research has shown that ER stress could induce lipolysis in adipose tissue, which contributes to an increase in circulating FFA and the accumulation of fat in other organs, such as the liver [107]. The ER plays a crucial role in various cellular functions, including protein synthesis and folding, lipid synthesis, and the formation of nascent lipid droplets [99,100]. Lipolysis under ER stress occurs in conjunction with elevated cAMP production and PKA activity, leading to increased phosphorylation of HSL, which in turn results in lipolysis in adipose tissue [107]. 

Evidence suggests that the cAMP signaling pathway is the main pro-lipolytic pathway in WAT. Catecholamines stimulate β-AR to activate Gs, and cAMP is stimulated by Gs proteins to generate PKA. PKA phosphorylation has two main targets, including HSL (the main lipase responsible for triglyceride hydrolysis) and lipid droplet-coated protein (PLIN). PLIN is a coating protein that adheres to the surface of lipid droplets [108]. In unstimulated adipocytes, PLIN acts as a barrier against lysis because it sits on the surface of lipid droplets and prevents HSL from interacting with lipid droplets [109]. PKA phosphorylates HSL at the site of the Ser 660, which is essential for its activation and translocation [110]. HSL activation thereafter fueled the catalyzation of TG hydrolysis into monoglycerides [111], whereby lipolysis occurs. Although alcohol exposure did not affect HSL and PLIN mRNA levels in mice, it markedly enhanced HSL serine 660 phosphorylation and elevated PLIN protein levels [87].

As a key protein in the modulation of fat mobilization [112], HSL is also regulated by other factors. In addition to being activated by cAMP/PKA phosphorylation in response to hormone stimulation, the enzyme can also be inactivated by protein phosphatase 2A (PP2A)-mediated dephosphorylation [113]. Methylation of PP2A by leucine carboxyl methyltransferase 1 (LCMT1) activates its function. In mice that were chronically fed an alcohol-containing diet, studies have demonstrated a decrease in the sialylation degree of the PP2A enzyme. This was indicated by a reduction in the S-adenosylmethionine/S-adenosylhomocysteine (SAM/SAH) ratio, ultimately resulting in uninhibited HSL activation and increased lipolysis. As a result, FFAs are released from adipose tissue at a higher rate [114]. 

Recently, the role of another lipase, ATGL, has also received much attention [115]. The substrate of ATGL is TG, which is the rate-limiting enzyme for lipolysis in adipose tissue [116]. Unlike HSL, ATGL is not directly phosphorylated by PKA, and its function depends on the activation of PLIN [117]. Evidence shows that alcohol exposure could increase the mRNA and protein levels of ATGL [88].

Alcohol exposure also inactivates adiponectin phosphatase 1 and upregulates phosphatase and tensin homolog (PTEN), and suppressors of cytokine signaling 3 (SOCS3) proteins in mice, leading to an increased release of FAs [88]. Therefore, hyperlipidemia represents a significant functional impairment in WAT resulting from chronic ethanol exposure.

As these enzymes, which are important in the process of lipolysis, are affected to varying degrees by alcohol, the above findings could partly explain the reduction in adipose tissue mass caused by alcohol.

#### 3.1.4. Insulin Signaling Pathway

The insulin signaling pathway is also an important pathway that affects lipolysis. Insulin inhibits lipolysis and promotes fat storage [118]. Insulin can stimulate phosphodiesterase-3B (PDE3B) activity by activating Akt and inhibit lipolysis by decreasing PKA. However, adipocytes obtained from rats fed a chronic alcohol diet did not exhibit any alterations in PDE3B activity, mRNA, or protein [32], whereas PDE3B activity was reduced in mice after intake of alcohol (20% w/v in drinking water) for 5 weeks, suggesting that the effects of this mechanism are not yet clear [119]. Insulin-induced phosphorylation of protein phosphatase 1 (PP1) leads to the subsequent dephosphorylation of HSL, thereby disrupting lipolysis [120,121]. Chronic alcohol intake inhibits PP1 phosphorylation [122], suggesting that alcohol may be a mechanism to overcome insulin-mediated inhibition of lipolysis. The results showed that both chronic and acute alcohol exposure enhanced the process of lipolysis, so the potentially enhanced insulin action observed in the chronic alcoholism group may inhibit lipolysis, but this inhibitory effect may be masked by other stimuli.

#### 3.1.5. PPAR Signaling Pathway

PPAR is predominantly distributed in WAT and plays a critical role in regulating adipose expansion and obesity [123]. Ethanol exposure downregulates the PPARγ gene in the adipose and reduces WAT mass, thereby inducing inflammation [124]. Through experimental studies, it has been observed that feeding mice with ethanol for a duration of 8 weeks led to a significant decrease in body weight. Measurements of adipocyte diameter indicated that the consumption of ethanol resulted in a significant reduction in the size of adipocytes [88]. Previous research has provided evidence that activating PPAR has a protective effect against alcoholic fatty liver disease. This activation has been found to stimulate the secretion of lipocalin and activate the hepatic lipocalin-SIRT1-AMP-activated protein kinase (AMPK) signaling pathway [125]. It was found that ethanol feeding significantly reduced PPAR-mRNA levels in adipose tissue, leading to adipose dysfunction and dysregulation of lipid homeostasis in the WAT liver axis, further promoting the development of alcoholic fatty liver [124]. AMPK is another important target of ethanol. The activation of AMPK is mediated through the metabolic modification and phosphorylation of a specific amino acid residue, Thr172, located within the alpha subunit of the AMPK protein [126]. Reduced activity of AMPK was observed after chronic-alcohol consumption, consistent with reduced phosphorylation of acetyl coenzyme A (acetyl-CoA) carboxylase (ACC) [127]. Due to the inhibitory effect of AMPK activation on lipolysis in adipose tissue, the decrease in AMPK levels associated with alcohol consumption may contribute to an increase in lipolysis. In addition, research has demonstrated that PPAR-γ is capable of trans-activating ATGL [128], with mRNA levels up-regulated four-fold in ethanol-fed mice [124]. At present, the expression of PPAR-γ and ATGL genes in WAT is controversial. The consumption of ethanol resulted in a reduction in the expression of PPAR-γ in WAT. However, it concurrently led to an increase in the expression of the ATGL gene. The result suggests that the expression of the ATGL gene appears to be regulated by a complex interplay of multiple factors. Insulin has a negative regulatory effect on the ATGL gene, and the elevation of ATGL mRNA levels in adipose tissue has been linked to insulin deficiency or insulin resistance [129].

#### 3.1.6. An Important Regulator: FGF21

FGF21 (fibroblast growth factor 21) has been identified as an additional regulator of adipose tissue lipolysis. It stimulates lipolysis independently of the catecholamine-activated pathway and inhibits lipid accumulation via PPARγ and C/EBP. FGF21, an energy-responsive adipokine, is exclusively secreted by WAT in response to feeding, glucose uptake, fatty acid synthesis, and activation of PPARγ [130]. The expression of FGF21 in plasma and epididymal white adipose tissue (eWAT) is upregulated in response to chronic binge alcohol consumption. Interestingly, in mice lacking FGF21, the alcohol-induced increase in lipolysis is effectively prevented. This suggests that FGF21 plays a crucial role in mediating the alcohol-mediated increase in lipolysis. In addition, alcohol-induced FGF21 knockout (KO) mice showed less reduction in eWAT mass compared with wild-type animals. Zhao et al. [86] concluded that in wild-type and non-KO mice, FGF21 promotes alcohol-induced lipolysis via activating the β-adrenergic pathway based on increased plasma acetylacetonate amine concentrations instead of insulin concentrations. As this directly contradicts the above findings [104], the role of FGF21 in alcoholism remains an area that needs further study, and its significance as a metabolic regulator is expected to continue expanding.

### 3.2. Alcohol Consumption and Lipid Synthesis in Adipose Tissue

In contrast to lipolysis, adipogenesis occurs during periods of excess energy in adipocytes. Although alcohol has a greater effect on lipolysis than on lipogenesis, it does regulate several parts of the lipogenesis pathway.

#### 3.2.1. Accumulation of Lipids

Adipocytes have two primary mechanisms for lipid accumulation. The first process occurs during normal daily feeding, where adipocytes uptake dietary lipids in the form of FFA from the circulation. This uptake is facilitated by the enzyme lipoprotein lipase (LPL) [131]. Adipocytes secrete LPL and transport it to nearby capillaries, catalyzing the hydrolysis of FFA by circulating triglyceride-containing lipoproteins [132,133,134]. These lipoproteins include chylomicrons (CMs), which are produced in the small intestine, and very low-density lipoprotein (VLDL), which is synthesized by the liver. The enzymatic action of LPL enables the release of FFAs from these lipoproteins for uptake and storage within adipocytes [34]. Additionally, adipocytes can take up glucose, which is converted into glycerol and used as subsequent esterification of FAs. The last stage in TG synthesis, the re-esterification of circulating FFAs, is mediated by diacylglycerol acyltransferase (DGAT) [135]. The second mechanism involved in lipid accumulation is de novo lipogenesis (DNL), which includes de novo synthesis of FAs from acetyl-CoA and esterification of these FAs into glycerol backbone to produce TGs. DNL can occur under fasting and feeding conditions [34]. By utilizing the DNL enzymes ACC1 and fatty acid synthase (FAS), this process converts acetyl-CoA to palmitate, which can subsequently undergo elongation and desaturation to form a variety of other FA species [136].

#### 3.2.2. LPL and VLDL

FA transport, FA, and TG synthesis are important functions of fat storage. Ethanol exposure stimulated lipolysis; however, functional analysis of exogenously fluorescently labeled FAs showed that feeding ethanol for 8 weeks significantly inhibited the ability of adipocytes to take up FAs [124]. The hydrolysis of TGs in TG-rich lipoproteins like CMs and VLDLs is primarily mediated by the enzyme LPL, and both LPL and the VLDL receptor are implicated in lipid uptake in adipose tissue [137]. The cluster of differentiation 36 (CD36) plays a vital role in regulating the uptake of FAs uptake at the plasma membrane of adipose tissue [88]. FAs hydrolyzed by LPL entered the cells in response to CD36. FATP1 and fatty acid binding protein 4 (FABP4) are both associated with FA transport. Studies have shown that ethanol feeding not only reduces LPL activity by 25% and 24% within two and four hours, but also downregulates genes associated with FA uptake and transport and reduces the mRNA expression of these transport-related enzymes, leading to reduced FA uptake and transport in adipose tissue [79].

#### 3.2.3. Glucose and AMPK/MEF2/GLUT4 Pathway

Glucose can act as another precursor substance for triglyceride synthesis in adipocytes. Glucose in the blood needs to enter the cells to be utilized, and glucose transporter protein 4 (GLUT4), a crucial protein, plays a pivotal role in facilitating glucose utilization within adipose tissue and skeletal muscle. GLUT4 is regulated by hypoxia, insulin, and exercise [138,139]. GLUT4 mRNA increases during muscle contraction or incubation with insulin in vitro [139]. We also found that, compared to endurance training alone, hypoxia after exercise training significantly increased the GLUT4 protein and mRNA in more muscles [138]. Inhibition of its expression and/or translocation can result in impaired utilization of glucose. Research by Minokoshi et al. shows [140] that chronic alcohol consumption reduces the expression of GLUT4 mRNA and protein levels in rat adipose tissue, which not only reduces glucose uptake by adipocytes, but is more likely a key step in the development of ethanol-induced insulin resistance. It is noteworthy that changes in GLUT4 expression in adipose tissue are believed to have a significant impact on systemic insulin sensitivity, potentially surpassing the influence of skeletal muscle and liver [141]. Insulin regulates GLUT4 through the insulin receptor substrate (IRS)-phosphatidylinositol-3-kinase (PI3K)-protein kinase B (Akt) pathway in adipose tissue. Early studies suggest that chronic alcohol feeding affects the tyrosine phosphorylation of PI3K and the phosphorylation of Akt in response to insulin stimulation [142,143]. Evidence suggests that chronic alcohol exposure may inhibit insulin action, but plasma insulin levels were not affected [88]. However, there is evidence for the opposite result, with alcohol intake at low doses increasing the phosphorylation of Akt [144], suggesting that insulin action is activated and thus Akt stimulates glucose uptake by GLUT4. This difference may be due to different patterns of alcohol exposure. 

In addition to insulin-PI3K-Akt, GLUT4 is regulated by other factors such as myocyte enhancer factor 2A (MEF2A) and MEF2D, which act as the promoters of GLUT4 transcription [145]. In one study, it was observed that chronic ethanol intake reduced the expression of MEF2A and MEF2D at both mRNA and protein levels in rat adipose tissue, which may contribute to the reduced expression of GLUT4 mRNA [146]. Further studies revealed that AMPK was shown to serve as an upstream regulator of MEF2 [147]. Activation of AMPK has been demonstrated to promote the expression of GLUT4 and facilitate basal translocation of GLUT4 in both skeletal muscle and adipocytes [148,149]. However, findings indicated that although feeding ethanol to rats did not noticeably affect overall AMPKα protein levels, it resulted in a reduced level of phosphorylated AMPKα (pAMPKα), suggesting that ethanol affected AMPKα activation. The inhibition of activation prevents AMPK from being transferred from the cytoplasm to the nucleus, which in turn affects the transcriptional level of MEF2 in the nucleus, ultimately causing a reduction in GLUT4 expression [147]. The above study proposed and confirmed the presence of the AMPK/MEF2/GLUT4 pathway in adipose tissue, where activated AMPK upregulates GLUT4 expression through MEF2. The inhibition of this pathway due to chronic ethanol consumption contributes, at least partially, to impaired GLUT4 expression in adipose tissue. This impairment subsequently leads to reduced insulin sensitivity, and compromised glucose tolerance, and potentially explains the observed decline in adipose tissue lipid synthesis.

Glucose serves as the immediate substrate for the subsequent conversion of FAs into glycerol-3-phosphate (glycerol-3-P) or acetyl-CoA. The transported glucose generates glyceraldehyde-3-phosphate (glyceraldehyde-3-P) in the glycolysis pathway. After that, glyceraldehyde-3-phosphate undergoes conversion to pyruvate, which then enters the tricarboxylic acid (TCA) cycle. The conversion of glucose to fatty acyl coenzyme A (FA-CoA) involves the activity of multiple enzymes, and the regulation of these enzymes is influenced by chronic alcohol consumption. Firstly, in rats and primary adipocytes from eWAT, alcohol reduces the phosphorylation of ATP citrate lyase (ACL), which enables the production of acetyl-CoA from citrate (a TCA cycle intermediate) [143]. Secondly, ACC catalyzes the transformation of acetyl-CoA into malonyl coenzyme A (malonyl-CoA). Alcohol also inhibited ACC Ser79 phosphorylation in vivo without change in vitro, and this change increased with alcohol dose [146].

#### 3.2.4. Transcription Factors: PPARγ and C/EBPα

Two important transcription factors, PPARγ and C/EBPα, regulate adipogenesis by binding to lipid synthesis-associated enzymes. Mechanisms by which alcohol impacts PPARγ and C/EBPα are included in Figure 2. Experimental results showed that chronic alcohol feeding down-regulated the expression of PPARγ and C/EBPα in eWAT by 43% and 74%, respectively [32]. The mitogen-activated protein kinase (MAPK) pathway plays a role in the regulation of PPARγ, and inhibiting MAPK activity can partially reverse the decreased PPARγ expression observed in rats exposed to alcohol. However, no changes were observed in alcohol-cultured 3T3-L1 cells [150]. Lipin1, another PPARγ regulator, also appeared to be reduced in the epididymal fat of chronically alcohol-fed rats, but again no changes were observed in cultured adipocytes [151].

Lipase fatty acid synthase (FASN), which is regulated by PPARγ, can catalyze the conversion of malonyl-CoA to FAs or FA-CoA (the active forms of FAs involved in metabolism) in preparation for the synthesis of triglyceride [151]. Furthermore, glycerol-3-P is formed via dihydroxyacetone phosphate (DHAP), which is transformed from glyceraldehyde-3-P. Glycerol-3-phosphate acyltransferase 3 (GPAT3) is a crucial enzyme that initiates the initial step of de novo TG synthesis [152,153]. GPAT3 catalyzes glycerol-3-P and FA-CoA to produce lysophosphatidic acid [154]. Subsequently, FA-CoA and LPA are catalyzed by 1-acylglycerol-3-phosphate acyltransferase (AGPAT) to produce phosphatidic acid. Lipin catalyzes the dephosphorylation of phosphatidic acid to produce DAG [155]. DGAT catalyzes the attachment of the last FA-CoA molecule to MAG or glycerol-3-P after which TGs are eventually formed. Lipase FASN and DGAT2 were significantly down-regulated in eWAT after long-term alcohol feeding, and the synthesis of DGAT1 and SREBP1c was also reduced, which is consistent with a reduction in tissue mass [124]. However, the expression of other lipid-producing enzymes, such as FASN, has also been reported to be unchanged in either cultured adipocytes or subcutaneous WAT (sWAT), suggesting that alcohol may exert a distinct effect on lipid metabolism for lipid deposition [151].

The impact of chronic alcohol exposure on eWAT and sWAT adipogenesis was observed in rats. The results revealed a significant decrease in eWAT mass, while there was no significant change in sWAT mass. Following chronic alcohol exposure, the expression levels of adipogenic enzymes and regulatory factors in sWAT did not change significantly [75,151].

Total protein levels of ADH and CYP2E1, two key enzymes in the oxidative metabolism of ethanol, were similar in both eWAT and sWAT. However, the expression levels of the aldehyde scavenging enzymes, aldehyde dehydrogenase 1A1 (ALDH1A1), ALDH2 and ALDH3A1 were higher in sWAT than in eWAT. This observation suggests that the higher expression of these enzymes in sWAT may contribute to its relative resistance to the detrimental effects of chronic alcohol exposure [156].

In vitro experiments clearly demonstrated that exposing eWAT or isolated 3T3-L1 adipocytes to acetaldehyde, a toxic metabolite of ethanol, significantly reduced the expression levels of PPARγ, C/EBPα, phosphorylated ACL (pACL), ACC, FASN, LPIN1, and DGAT2. It was confirmed that different levels of aldehyde metabolizing enzymes in eWAT and sWAT result in different chronic alcohol exposure responses. In addition, the use of the ALDH inhibitor cyanamide further reduced some lipid-producing enzymes and regulatory factors, but the exposure of 3T3-L1 adipocytes to ethanol did not significantly alter the protein levels of lipid-producing enzymes and regulatory factors [157].

Overall, while the effects of chronic alcohol intake on regulators of adipogenesis may differ between model systems (in vivo or in vitro), most findings support the possibility that alcohol induces reductions in several regulatory proteins. Whether these reductions lead to an overall reduction in adipogenesis is unclear, but injections of labeled TG in chronically alcohol-fed rats did not observe significant changes in triglyceride synthesis rates [124].

### 3.3. The Impact of Alcohol Consumption on Other Aspects

Apart from acting on lipolysis and lipid synthesis, alcohol also impacts the secretion of adipokines, including ADIPO, LEP, and omentin, which all play important physiological functions. There are few studies on omental protein, whose function is to increase insulin sensitivity of adipocytes. We just know that excessive alcohol consumption will cause an increase in its concentration in the plasma of people with ALD [28]. Furthermore, ethanol also affects the oxidative decomposition of fatty acids to generate heat. 

#### 3.3.1. ADIPO

ADIPO, a 30 kDa adipokine, is primarily secreted by adipocytes and exhibits anti-diabetic, anti-atherosclerotic, anti-inflammatory, and cardioprotective properties, making it a valuable therapeutic molecule [158]. Chronic ethanol exposure enhances the synthesis of CYP2E1, an enzyme responsible for ethanol metabolism, and simultaneously decreases its degradation in the liver [159]. Chronic ethanol intake did not increase oxidative stress and lipid peroxidation in the livers of CYP2E1 (−/−) mice [60,160], suggesting that CYP2E1 may contribute to the damage caused by ethanol exposure. Although CYP2E1 is primarily expressed in the liver, there is also a detectable expression of CYP2E1 in adipose tissue at relatively lower levels [90].

After long-term exposure to ethanol, there was a reduction observed in circulating ADIPO levels [161]. It was found that ethanol-induced the expression of CYP2E1 in rat epididymal adipose tissue, and mediated ROS indirectly led to the decrease of ADIPO secretion [162]. Subsequent evidence showed that ethanol feeding did not reduce intracellular ADIPO, but affected the secretion of ADIPO. ADIPO undergoes post-translational modifications within the endoplasmic reticulum (ER) prior to its secretion into the extracellular space [158]. Maintaining a balanced redox state in the ER and Golgi apparatus is crucial for ensuring accurate post-translational modifications of proteins [163]. Measurement of the glutathione/oxidized glutathione (GSH/GSSG) ratio serves as a valuable approach to assessing ER functionality. In the case of high-density microsomes obtained from rats exposed to ethanol, a reduced GSH/GSSG ratio of 2.9:1 was observed, indicating an elevated level of oxidative stress following ethanol consumption [162]. The increased oxidative stress was associated with the up-regulated expression of CYP2E1, as demonstrated by the presence of 4-hydroxynonenal (4-HNE) protein adducts in adipocytes and an elevation in ADIPO carbonylation [163,164,165,166]. While overexpression of CYP2E1 in 3T3-L1 adipocytes did not affect intracellular ADIPO concentration [161], ethanol culture did lead to the inhibition of ADIPO release into the extracellular medium. These results indicated that ethanol decreased the ADIPO secretion of 3T3-L1 adipocytes that overexpressed CYP2E1 [162].

#### 3.3.2. LEP

LEP, a 16-kDa cytokine-type peptide hormone, is predominantly released by adipocytes. It serves as a key regulator of food intake and energy balance by interacting with its receptor, LEP receptor (LEPR) [167,168]. LEP can activate LEPR signals in the hypothalamus via multiple pathways. Among them, Janus tyrosine kinase 2/signal transducer and activator 3 (JAK2/STAT3) pathway, is regarded to be crucial to mediate the action of LEP in energy regulation. Under the stimulation of LEP, STAT3 phosphorylates. Phosphorylated STAT3 (pSTAT3) exerts the physiological function of LEP by combining and regulating its target gene [169]. Recent findings have indicated that ethanol suppresses the activation of the JAK/STAT pathway, which is typically triggered by certain members of the class I cytokine receptor family. The experimental findings indicate that alcoholics exhibit significantly higher levels of LEP, and interestingly, ethanol inhibits the activation of the STAT3 pathway induced by LEP. Furthermore, ethanol has been observed to influence the effects of LEP in both peripheral tissues and the central nervous system [168,170].

#### 3.3.3. Thermogenesis

Lipids serve as a major source of cellular energy, alongside glucose. Hepatic FA β-oxidation is a crucial process involved in energy production. The carnitine acyl transferase (CAT) system transports long-chain FAs to mitochondria, in which carnitine palmitoyltransferase 1 (CPT1) serves as the key regulatory enzyme [170,171]. Hepatic CPT1 activity is inhibited and enzyme sensitivity to malonyl-CoA inhibition is increased after alcohol feeding [172]. Acyl-CoA dehydrogenase (ACD) represents the central activities in mitochondrial β-oxidation [173]. Long-chain, medium-chain, and short-chain FAs have separate ACDs [174,175]. Acyl-CoA is then converted to acetyl-CoA regulated by ACD. Ethanol feeding influences thermogenesis by inhibiting ACD gene expression [143].

## 4. Alcohol Consumption and BAT

BAT differs markedly in morphology and function from WAT. BAT is characterized by the presence of multiple small lipid droplets arranged in multiple chambers, accompanied by a central nucleus. In contrast, WAT typically consists of a single large lipid droplet, housed in a single chamber, with a nucleus located towards the periphery. A distinguishing feature of BAT is its abundance of mitochondria, which contribute to its heightened metabolic rate and thermogenic capabilities. Notably, alcohol has been found to readily permeate BAT, as demonstrated in studies involving mice exposed to alcohol [176]. The activity of ADH in BAT was found to be lower compared to that in the liver, and it did not show any significant change following a 10-day period of chronic alcohol consumption [177]. 

The effect of alcohol on the quality of BAT in the interscapular warehouse is inconsistent because, according to different experimental conditions, the result may be no change, increase, or decrease [178]. After subjecting male mice to 10 days of alcohol feeding [179], male rats to 14 days of alcohol feeding [180], or mice to 5 weeks of alcohol feeding [119], no significant alteration in BAT weight was observed. However, a reduction in BAT weight was noted in male mice after 25 days of alcohol exposure [179]. When obese mice with a body weight exceeding 40 g were provided with water containing alcohol for a duration of 5 weeks, it was observed that the expected increase in BAT did not occur [119]. This indicates that alcohol intake could impede the expansion of BAT during malnutrition. It is worth noting that a sufficient duration of alcohol intake is necessary to induce adaptive responses in BAT.

Similar to its effects on WAT, alcohol regulates lipolysis and lipogenesis within BAT. Both acute and chronic alcohol consumption generally leads to a reduction in the activity of HSL, an enzyme responsible for the breakdown of fats, in BAT and primary BAT adipocytes [176]. In addition, acute and chronic alcohol also consistently reduced cAMP accumulation, suggesting inhibition of lipolysis by the β-AR pathway. These findings indicate that the decrease in lipolysis induced by alcohol in BAT may contribute to the preservation of BAT tissue mass, despite reports of reduced activity of the lipogenic enzyme LPL in BAT with chronic alcohol consumption. On the other hand, acute alcohol exposure does not seem to affect LPL activity in vivo and in vitro, suggesting that longer-term alcohol exposure may be necessary to observe effects on LPL activity [180].

The thermogenesis of BAT is dependent on lipolysis and the availability of FFAs, which can be utilized as a fuel source or activate UCP1 [181,182]. Additionally, BAT plays a crucial role in lipid clearance from the bloodstream, similar to the liver. Therefore, alterations in WAT due to alcohol-induced lipolysis may also impact BAT. Chronic alcohol increases the lipolysis of WAT, which will enhance the utilization and uptake of FFA by BAT, thereby improving the thermogenic capacity of BAT [183]. In individuals with obesity, this effect may be beneficial. However, in conditions typically associated with malnutrition, the alcohol-induced increase in thermogenesis may contribute to weight loss and potentially worsen overall health status [184]. However, whether BAT absorbs FAs from WAT during chronic alcohol intake remains to be determined, which highlights an interesting area for future research.

In summary, the impact of alcohol on BAT is unclear, but our understanding of the functional properties of BAT is significantly improved compared to what was previously available, which could help further research. As the interscapular BAT only constitutes a minor portion (about 20–25%) of the overall BAT, further investigations should be extended to encompass female BAT and other BAT depots. Additionally, studies should be directed toward assessing the potential thermogenic effects of alcohol and their potential implications.

## 5. Effect of Alcohol on Adipose Tissue–Liver Axis

Recent studies highlight the significant contribution of “the adipose tissue-liver axis” in the development of ALD. The adipose tissue–liver axis refers to the two-way relationship between adipose tissue and the liver by regulating lipid metabolism. Coordination of lipid metabolism between WAT and the liver is the key to maintaining lipid homeostasis [185,186]. Studies show that lipid homeostasis will be disrupted by chronic alcohol consumption in the adipose tissue–liver axis [78]. Chronic alcohol consumption triggers off lipolysis of the WAT and causes excessive release of FAs. FAs are delivered to the liver and stored as TGs, resulting in alcoholic fatty liver, the primary and most prevalent pathological form of ALD [187]. Studies consistently indicate that decrease secretion of ADIPO in adipose tissue and the impaired expression of ADIPORs in the liver are one of the pathogeneses of alcoholic hepatic steatosis. ADIPO, when present in circulation, exerts its biological functions via binding to ADIPOR2, which is mainly expressed in the liver. The activated ADIPO signaling stimulates the AMPK pathway, which regulates liver lipid metabolism via concurrently repressing DNL and facilitating β-oxidation of FAs [188]. In addition, extensive research has highlighted the impact of altered gene expression in adipocytes on liver functions such as lipid metabolism, inflammation, and regeneration, demonstrating the interaction between adipose tissue and the liver [28]. When the growth hormone in mice increases, the deletion of Jak2 can significantly inhibit the accumulation of neutral lipids and serum hepatocyte damage markers by reducing the rate of lipolysis, which has a significant protective effect on hepatocyte damage and can improve fatty liver to a certain extent [189]. New findings suggest that when fumarate hydratase is deficient specifically in adipose tissue, it can help reduce liver steatosis in mice [190]. It is reported that adipocyte-specific Fsp27 disruption could inhibit obesity induced by a high-fat diet by promoting impaired fat storage function and increased lipolysis rate [191]. Deletion of specific genes in hepatocytes can simultaneously influence adipogenesis, lipolysis, and inflammation in adipose tissue [28]. Matsusue et al. [192] found that the liver-specific deletion of PPARγ in leptin-deficient mice resulted in reduced insulin sensitivity. Some studies suggest that high-fat diet-induced increased WAT mass is suppressed by liver-specific deletion of the NF-κB essential modulator gene [192]. Experiments showed that growth hormone-mediated lipolysis increased due to hepatocellular specific deletion of Jak2 reduced body fat and increased plasma FFA levels [193]. Alcohol regulated the crosstalk between adipose tissue and the liver, but existing research mainly focused on studying the individual effects of alcohol intake on simple adipose tissue or the liver. More research is expected to gain a comprehensive understanding of the role of alcohol in the dynamic interaction between adipose tissue and the liver. Future research should aim to bridge this knowledge gap and provide insights into the complex mechanisms underlying this crosstalk.

## 6. Conclusions

Alcohol consumption has multifaceted effects on overall health, involving complex interactions among different organs and tissues. Adipose tissue has emerged as a key player in the development and progression of ALD. The impact of ethanol on the immune response and metabolic functions of adipose tissue contributes to tissue injury and influences the regulation of alcohol consumption. Significant advances have been made in unraveling the role of adipose tissue in alcohol-related damage. However, the precise molecular mechanisms underlying the interplay between adipose tissue injury and the progression of liver disease caused by alcohol consumption remain to be fully understood. Further research is necessary to shed light on these mechanisms and provide a deeper understanding of the complex relationship between adipose tissue and liver health in the context of alcohol-related conditions.

## Figures and Tables

**Table 1:** Effects of alcohol consumption on various factors in adipose tissue of rodents and humans.

Factors	Effects on Adipose Tissue	Changes with Alcohol Abuse (Plasma Chronic Alcohol)	
		Rodent	Human
Adiponectin	Sensitizes to insulin, increases adipocyte mass and reduce adipose inflammation	↓ [79]	↑ [80]
Leptin	Suppression of appetite, promotion of energy expenditure	↑ [81]	↑ [82]
Resistin	Stimulate lipolysis and FA release, suppress adiponectin	↑ [83]	↑ [84]
Chemerin	Adipogenesis and adipocyte differentiation	↑ [85]	↑ [85]
NE/EP	Important regulators of lipolytic activity	↑ [86]	NR
FGF21	Inhibits lipid accumulation	↑ [87]	NR
IL-6, TNF-α	Inflammatory cytokines	↑ [78]	↑ [59]
Adipose tissue mass and adipocyte size	—	↓ [88]	↓ (↑ in VAT and ↓ in SAT) [89]
Triglyceride degradation	—	↑ [76]	NR
Pathogenic effects		Insulin resistance [78]	Glucose intolerance [90]

EP, epinephrine; FGF21, fibroblast growth factor 21; IL-6: interleukin-6; NE, norepinephrine; NR, not reported; SAT, subcutaneous adipose tissue; TNF-α, tumor necrosis factor alpha; VAT, visceral adipose tissue.

## Data Availability

Not applicable.
